# Bridging the Gap Between Strategy and Implementation in Antimicrobial Stewardship: Insights from the ARCO Step II Interregional Delphi Consensus in Northeastern Italy

**DOI:** 10.3390/microorganisms14081811

**Published:** 2026-08-17

**Authors:** Paola Anello, Giacomo Berti, Edoardo Miotto, Umberto Gallo, Stefano Palcic, Chiara Roni, Giulia Dusi, Michele Chittaro, Massimo Crapis, Vincenzo Baldo, Daniele Mengato, ARCO Working Group

**Affiliations:** 1 Hospital Medical Management, ULSS 2 Marca Trevigiana Health Authority, St. Valentino Hospital, 31044 Montebelluna, Italy; paola.anello@aulss2.veneto.it (P.A.); edoardo.miotto@aulss2.veneto.it (E.M.); 2 Project and Clinical Research Unit, University-Hospital of Padua, via Giustiniani, 2, 35128 Padua, Italy; 3 Department of Cardiac Thoracic and Vascular Sciences and Public Health, University of Padua, via Giustiniani, 2, 35128 Padova, Italy; vincenzo.baldo@unipd.it; 4 Primary Care Pharmaceutical Departments, ULSS 6 Euganea Health Authority, Via Enrico degli Scrovegni, 12, 35131 Padova, Italy; umberto.gallo@aulss6.veneto.it; 5 Farmaceutica Territoriale, Azienda Sanitaria Universitaria Integrata Giuliano-Isontina (ASUGI), 34142 Trieste, Italy; stefano.palcic@asugi.sanita.fvg.it; 6 Hospital Pharmacy Unit, Cattinara Hospital, Azienda Sanitaria Universitaria Giuliano Isontina, 34149 Trieste, Italy; chiara.roni@asugi.sanita.fvg.it; 7 Hospital Pharmacy Unit, ASST del Garda, 25015 Desenzano del Garda, Italy; giulia.dusi@asst-garda.it; 8 Hospital Health Direction, ASFO ‘Santa Maria degli Angeli’ Hospital of Pordenone, 33170 Pordenone, Italy; michele.chittaro@asfo.sanita.fvg.it; 9 Infectious Diseases Unit, Azienda Ospedaliero-Universitaria di Ferrara, 44124 Ferrara, Italy; massimo.crapis@ospfe.it; 10 Hospital Pharmacy Unit, Azienda Ospedale-Università, via Giustiniani, 2, 35128 Padova, Italy

**Keywords:** antimicrobial stewardship, Delphi Consensus, healthcare governance, Regional Health Policy, Clinical Pharmacist

## Abstract

National and international frameworks provide strategic direction for antimicrobial stewardship (AMS), while their translation into operational practice remains inconsistent. Building on ARCO Step I findings, this study aimed to establish an interregional, multidisciplinary consensus on sustainable AMS implementation strategies across hospital and community settings in Northeastern Italy. A modified Delphi methodology engaged 33 interdisciplinary professionals from Northeastern Italy. Eighteen operational statements across three domains (Governance, Hospital AMS, Community AMS) underwent two quantitative scoring rounds (relevance and feasibility; 1–10 scale) separated by a structured in-person workshop with thematic round-table discussions. Consensus threshold was defined as median score ≥ 8.0; high-priority statements required combined score ≥ 8.5. All 18 statements achieved consensus for relevance, whereas 12 statements achieved consensus for feasibility. Post-discussion median scores feasibility showed a numerically larger increase for feasibility (Δ +0.78) compared to relevance (Δ +0.44), indicating that structured deliberation reduced perceived implementation barriers. Furthermore, 14 statements were classified as high-priority. Qualitative analysis identified key enablers (CEO performance mandates, multidisciplinary teams, standardized indicators) and barriers (digital infrastructure gaps, resource constraints). The participatory approach helped translate strategic policy into a set of practice-oriented recommendations, offering a potentially transferable model for regional health authorities implementing AMR action plans, pending evaluation of actual implementation.

## 1. Introduction

Antimicrobial resistance (AMR) continues to represent one of the most serious global health challenges, threatening the effectiveness of modern medicine and the sustainability of healthcare systems. In Europe, AMR is estimated to cause approximately 35,000 deaths each year, with Italy accounting for more than 10,000 of these—over one third of the total burden across the EU [1,2,3].

Antimicrobial Stewardship (AMS) and Infection Prevention and Control (IPC) programs are widely recognized as essential strategies to contain AMR and optimize antibiotic use across all levels of care. Their implementation, however, is highly dependent on the governance and organizational context in which they are embedded [4,5].

While international frameworks such as the WHO Core Elements for Hospital AMS and the European One Health Action Plan outline key principles, the translation of these strategies into practice remains uneven. Studies have shown that variability in leadership commitment, team composition, data accessibility, and accountability mechanisms explains much of the difference in AMS performance across healthcare systems [6].

In Italy, the National Action Plan on Antimicrobial Resistance (PNCAR) 2022–2025 provides a strategic framework for integrating AMS and IPC activities [7]. However, the Italian health system is strongly decentralized: the 21 regions and autonomous provinces hold substantial autonomy in planning and organizing healthcare services, which allows contextual adaptation but also produces marked variability in AMS implementation and resource allocation across regions, making regional-level operationalization of steward-ship strategies key to national coherence [8].

Within this context, the Veneto Region has played a pioneering role, formalizing a three-tier AMS/IPC governance model (Regional Multidisciplinary Group, Hospital Multidisciplinary Teams, and Community Multidisciplinary Teams) under Regional Decree No. 1402/2019 [9].

Building upon this model, the ARCO Project (Approcci di Rete per il Contrasto all’Antimicrobico Resistenza Ospedale–Territorio) was initiated in 2023 to evaluate and strengthen AMS governance across healthcare organizations. The first phase, ARCO Step I, combined a region-wide survey with a multidisciplinary focus group, revealing good alignment with regional directives but also persistent gaps in multidisciplinary collaboration, uneven resource distribution, and limited integration between hospital and community AMS programs [10]. These findings underscored the need to move from descriptive assessment to operational consensus, identifying practical strategies to reinforce stewardship at system level.

To address this need, ARCO Step II expanded the initiative to an interregional scale, engaging professionals from Veneto, Friuli Venezia Giulia, and Trentino-Alto Adige in a structured Delphi-based consensus process. The goal was to translate the strategic directions identified in Step I into shared operational statements, validated through multidisciplinary discussion and interregional benchmarking. This participatory approach was designed to bridge the gap between policy and implementation, generating context-sensitive solutions that could inform regional and national AMS governance frameworks.

The objectives of the ARCO Step II study were therefore: first, to establish a multidisciplinary, interregional consensus on key strategies for the sustainable implementation of AMS across hospital and community settings; second, to assess the perceived relevance (strategic importance) and feasibility (implementability) of 18 operational statements developed by the ARCO Steering Committee using a modified Delphi methodology; third, to identify high-priority actions that can serve as a shared roadmap for regional health authorities and healthcare organizations to advance AMS governance and practice in alignment with national and European strategies.

## 2. Materials and Methods

The ARCO Step II initiative was conducted as a multicenter, inter-regional consensus study employing a modified Delphi methodology to identify and prioritize AMS implementation strategies. The design utilized a sequential mixed-methods approach, combining two rounds of quantitative scoring with an intermediate qualitative deliberation phase to enhance the perceived feasibility of complex system-level statements. This study is reported in accordance with the ACCORD (ACcurate COnsensus Reporting Document) guideline for consensus-based studies in biomedicine; the completed checklist is provided as Supplementary Table S2.

In 2024, a multidisciplinary Steering Committee was established to guide the design and methodological framework of the ARCO Step II study. The Committee was composed of six professionals from the Veneto and Friuli Venezia Giulia Regions, all actively engaged in AMR and stewardship initiatives within their regional health systems. Specifically, the group included two medical directors, two hospital pharmacists, and two territorial pharmacists, reflecting complementary expertise in governance, clinical practice, and pharmaceutical stewardship across hospital and community settings. Committee members were identified through a professional network of experts involved in AMS governance and policy implementation at regional level.

Working collaboratively, the Steering Committee defined the conceptual framework of the ARCO Step II study and translated the findings of ARCO Step I into a structured set of operational proposals suitable for interregional validation. Drawing on the scientific literature, national and regional policy frameworks (including the PNCAR 2022–2025 [7] and Regional Decree No. 1402/2019 [9]), and the empirical evidence from ARCO Step I [10], the group formulated 18 preliminary operational statements addressing key aspects of AMS governance and implementation. Operational parameters embedded in individual statements were sourced differently depending on their basis: for example, the interoperability and data-governance requirements of Statement 1.5 and the AWaRe-based prescribing alerts of Statement 3.3 draw directly on WHO and EU-JAMRAI guidance [1], whereas time-bound operational thresholds without a single published source reflect Steering Committee judgment informed by participants’ organizational experience rather than a specific published standard. The statements were developed and iteratively refined within the Steering Committee.

To facilitate structured discussion during the consensus process, the statements were organized into three thematic domains: Governance and Organization (1.1–1.6), strategic and managerial enablers for AMS integration; Hospital AMS Implementation (2.1–2.6), operational and clinical processes in acute care settings; Community and Primary-Care AMS Approaches (3.1–3.6), stewardship actions and coordination mechanisms in primary and territorial care. These statements formed the basis for the Delphi-based interregional consensus workshop held in May 2025.

**Figure 1:**
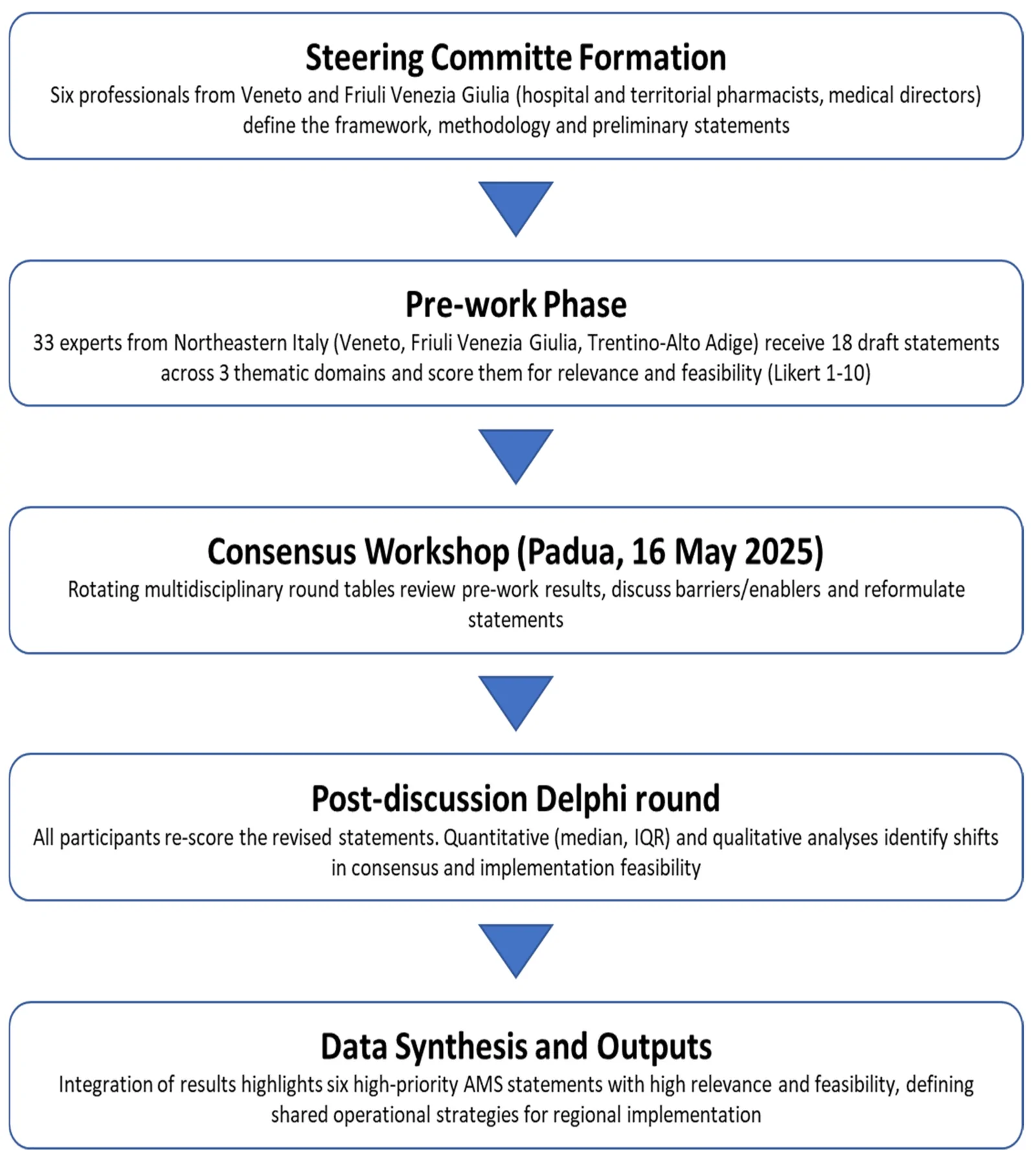
Flowchart illustrating the design and methodological phases of the ARCO Step II project.

Consensus was achieved through a structured Delphi-based process, a well-established methodology for developing expert consensus in healthcare (Figure 1). The Steering Committee designed the process to combine quantitative scoring with qualitative, face-to-face discussion, ensuring both analytical rigor and practical insight.

Experts were identified by the Steering Committee through the existing regional AMS and IPC professional networks of the three participating regions and were invited to participate on a voluntary basis, with no financial or other incentive offered. A multidisciplinary panel of 33 professionals from the three Northeastern Italian regions—Veneto, Friuli Venezia Giulia, and Trentino-Alto Adige—participated in the consensus, representing the full spectrum of AMS and IPC expertise: infectious disease specialists (n = 8), clinical microbiologists (n = 5), hospital pharmacists (n = 8), territorial/district pharmacists (n = 5), medical directors (n = 4), a general practitioner (n = 1), and public health/governance professionals (n = 2).

Each participant was actively engaged in AMS and/or IPC governance. The first Delphi round (pre-work phase) involved independent scoring of 18 preliminary statements for relevance (strategic importance) and feasibility (practical feasibility) using two separate ten-point Likert scales (1 = minimum, 10 = maximum).

Scores and comments were collected anonymously and served as the baseline for discussion. An in-person consensus workshop was then held on 16 May 2025 in Padua, during which participants rotated across three thematic round-table sessions—Governance, Hospital AMS, and Community AMS—each co-moderated by members of the Steering Committee. Discussions focused on identifying operational enablers, barriers, and context-specific adaptations for each statement. A medical writer documented the proceedings in real time, capturing key arguments and proposed revisions. When needed, statements were reformulated through open discussion, and agreement on the revised text was reached by group consensus. Following the workshop, a second Delphi round (post-discussion phase) was conducted during the final plenary session. Participants anonymously re-scored all revised statements for both relevance and feasibility using the same ten-point scale. Quantitative results were subsequently analyzed to assess convergence in median scores between rounds, while qualitative notes were synthesized thematically to contextualize the consensus process and capture the rationale underlying final prioritizations.

The Steering Committee established consensus thresholds a priori, following established Delphi methodology principles [11]. A median ≥ 8.0 on a 10-point scale was chosen because it lies within, and toward the upper end of the wide range of consensus thresholds (50–97% of the maximum score) reported across published Delphi studies, and it exceeds the 70–75% agreement most commonly adopted [11,12]. A statement was considered to have achieved consensus if the median score was ≥8.0 for both relevance and feasibility dimensions. Strong agreement between participants was defined as an interquartile range (IQR) ≤ 2.0, indicating limited variability across respondents. Statements were then stratified by implementation priority: those with a combined score ≥ 8.5, calculated as the arithmetic mean of median relevance and feasibility, were designated as high-priority actions, suitable for immediate operationalization within regional health systems.

Data from both the quantitative and qualitative phases were analyzed to evaluate the level of consensus and to interpret the rationale underlying score variations between rounds.

For quantitative data, responses from Rounds 1 and 2 were analyzed descriptively. For each statement, the median and interquartile range (IQR) were calculated to assess the degree of agreement and the measurable change in relevance and feasibility scores between rounds. The combined score was calculated as the arithmetic mean of relevance and feasibility medians for each statement. Scores were collected and reported anonymously at each assessment round, and no participant identifiers were retained to enable linkage of observations across rounds. Therefore, the dataset consisted of independent cross-sectional summaries rather than repeated individual measurements, precluding paired and other longitudinal inferential analyses. Statistical analyses were performed using R version 4.5.0 [13]. For qualitative data, documentation from the three round-table sessions—including moderators’ notes and verbatim transcripts synthesized by the medical writers—was compiled into a comprehensive report. These materials were then subjected to thematic analysis by the Steering Committee to identify recurrent themes, organizational barriers, enabling factors, and the rationale behind shifts in feasibility perception.

## 3. Results

### 3.1. Delphi Rounds and Scoring Evolution

Eighteen statements were assessed across three predefined thematic domains: Governance and organization (1.1–1.6), Hospital AMS implementation (2.1–2.6), and Community and primary-care AMS implementation (3.1–3.6) (Table 1).

**Figure 2:**
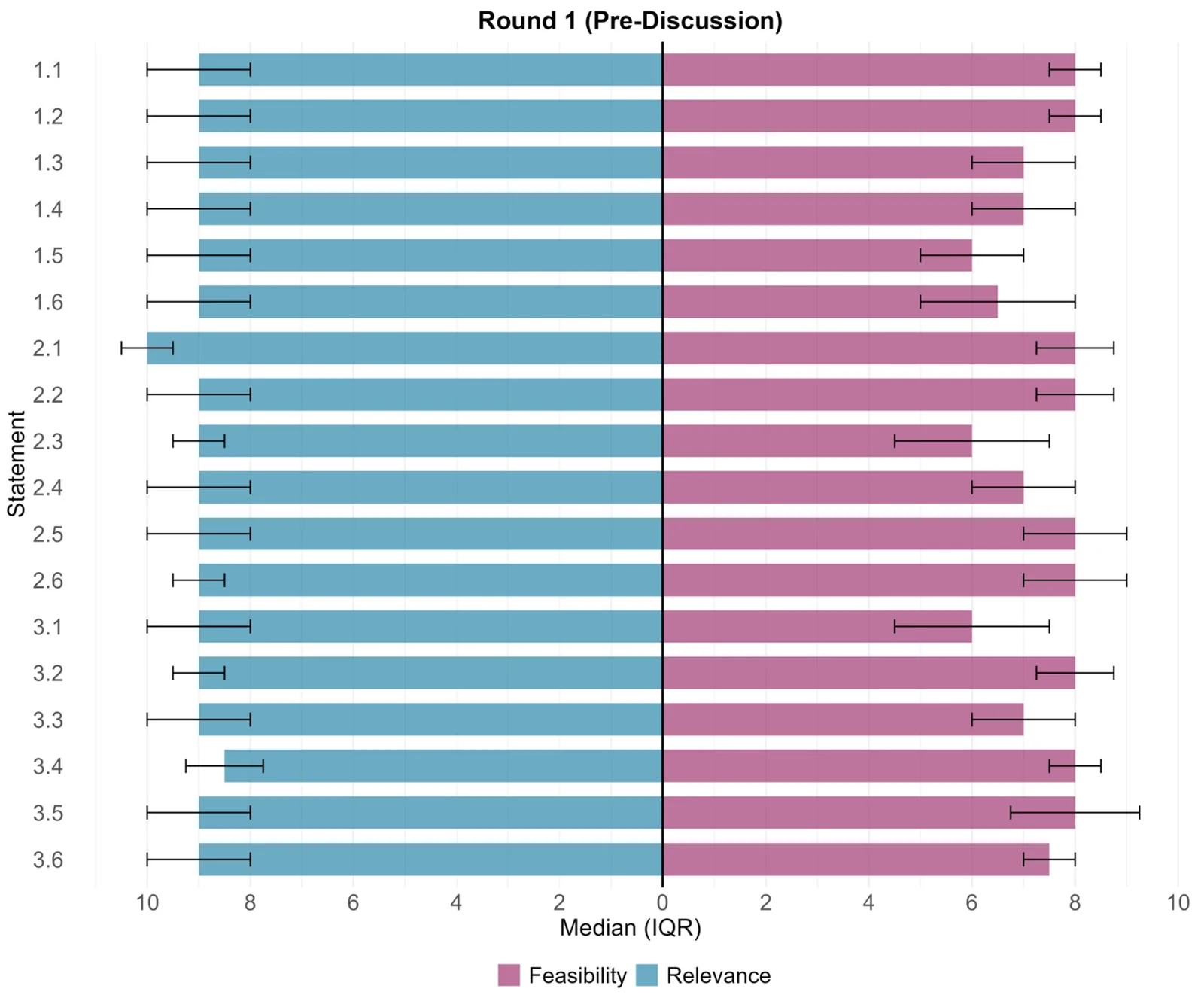
Tornado plot of Round 1 (pre-discussion) median scores for relevance (blue) and feasibility (pink) for each of the 18 statements, grouped by domain (1 = Governance and organization; 2 = Hospital AMS implementation; 3 = Community and primary-care AMS implementation). Bars show the median score (0–10 scale); horizontal whiskers show the interquartile range (IQR).

In Round 1 median relevance scores were consistently high across all statements (range 8.5–10.0), confirming the shared perception of the strategic importance of the proposed actions. Conversely, feasibility scores were initially more variable (range 6.0–8.0), reflecting the heterogeneity of healthcare settings and organizational resources across the three participating regions (Table 2, Figure 2).

**Figure 3:**
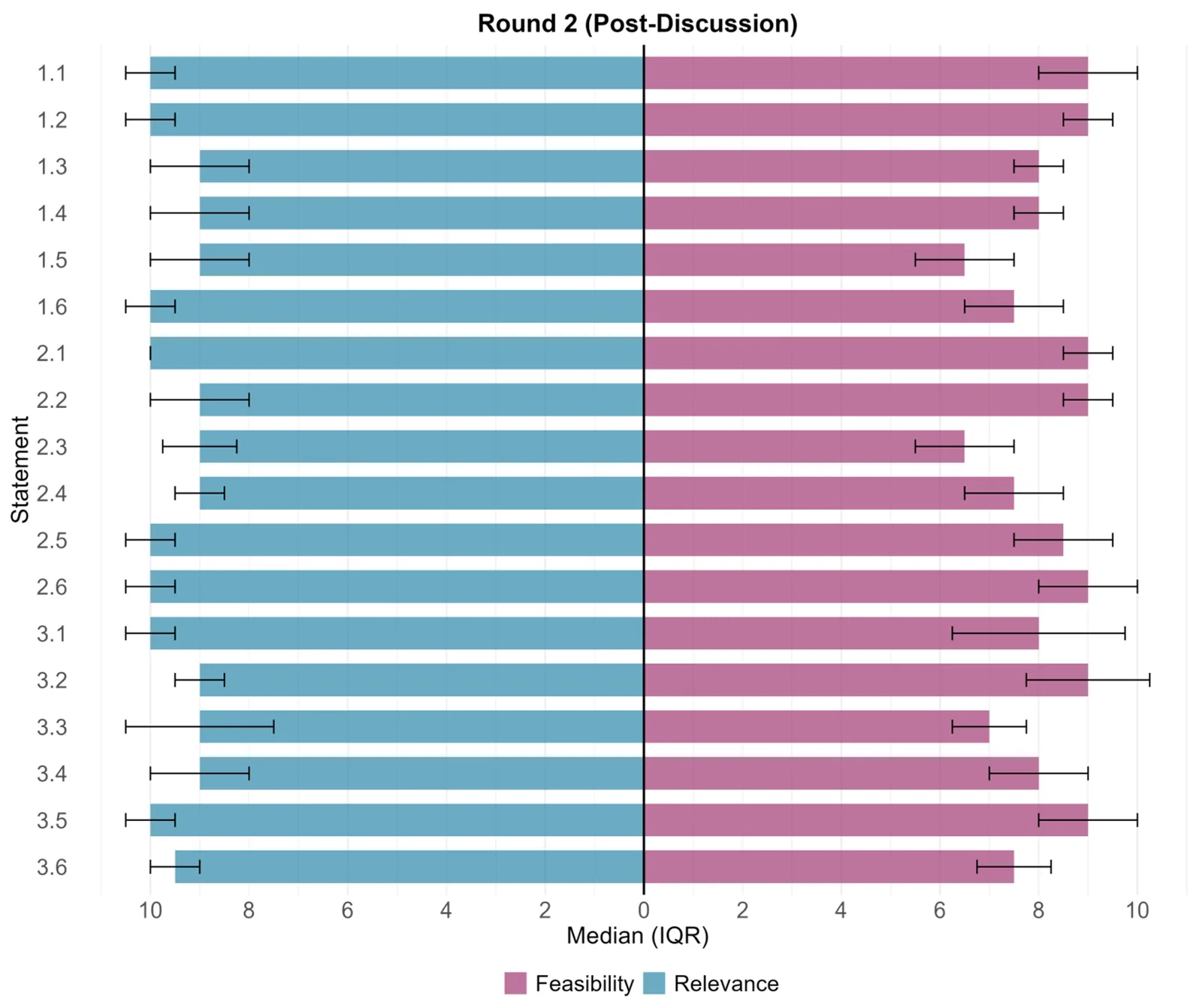
Tornado plot of Round 2 (post-discussion) median scores for relevance (blue) and feasibility (pink) for each of the 18 statements, grouped by domain (1 = Governance and organization; 2 = Hospital AMS implementation; 3 = Community and primary-care AMS implementation). Bars show the median score (0–10 scale); bars show the median score and whiskers show the IQR.

After the workshop discussions—Round 2—feasibility increased for fifteen statements, with a mean gain of +0.78 points (SD = 0.49), while relevance improved slightly (mean Δ = +0.44, SD = 0.48). The improvement was most evident for governance-related and territorial statements, suggesting that interdisciplinary discussion and interregional exchange helped clarify operational pathways and context-specific enablers. The mean of median relevance scores was 9.47 (SD = 0.50), with eight statements with a median score of 10.0. By contrast, the mean of median feasibility scores was 8.11 (SD = 0.88), with no statement reaching a median score of 10.0 (Table 2, Figure 3).

Strong agreement (IQR ≤ 2.0) was observed for 33 out of 36 evaluations (92%), considering both relevance and feasibility dimensions across all statements. Only three evaluations showed wider dispersion: statement 3.3 (digital prescribing alerts for GPs) for relevance, and statements 3.1 (territorial AMS teams) and 3.2 (standardized community procedures) for feasibility. Notably, all three exceptions pertained to the community and primary-care domain, suggesting greater heterogeneity in expert perceptions regarding territorial stewardship strategies (Table 2).

Twelve out of 18 statements (68%) achieved full consensus, defined as median ≥8.0 for both relevance and feasibility: 1.1–1.4, 2.1, 2.2, 2.5, 2.6, 3.1, 3.2, 3.4, and 3.5. The remaining six statements (1.5, 1.6, 2.3, 2.4, 3.3, 3.6) did not reach consensus solely due to insufficient feasibility scores (median < 8.0), while all maintained high strategic relevance (median ≥ 8.0) (Table 2).

Fourteen statements reached a combined median score ≥ 8.5; therefore, they were designated as high-priority recommendations: 1.1–1.4, 1.6, 2.1, 2.2, 2.5, 2.6, 3.1, 3.2, 3.5 and 3.6. Fourteen statements reached a combined median score ≥ 8.5; therefore, they were designated as high-priority recommendations: 1.1–1.4, 1.6, 2.1, 2.2, 2.5, 2.6, 3.1, 3.2, 3.4, 3.5 and 3.6. It should be noted that statements 1.6 and 3.6 reached this combined threshold despite feasibility medians of 7.5, which did not independently meet the ≥8.0 feasibility consensus criterion. These statements define a coherent strategic–operational core linking governance accountability, multidisciplinary coordination, and the systematic use of data for continuous improvement (Table 2).

### 3.2. Qualitative Insights from the Discussions

The qualitative analysis revealed strong consensus on the strategic importance of antimicrobial stewardship across all organizational levels, while highlighting considerable variability in implementation readiness and resource availability.

Governance and structural enablers were identified as foundational prerequisites for effective AMS implementation. Participants unanimously endorsed the integration of AMS objectives into CEO performance mandates and regional evaluation systems as critical mechanisms for institutional accountability. Digital infrastructure emerged as both a strategic priority and a major challenge, with participants reporting wide variability in digital maturity and persistent lack of interoperability between hospital and territorial databases.

Hospital AMS implementation generated consensus on core components while revealing feasibility gaps. Multidisciplinary teams, updated antibiotic formularies, and regular reporting were considered essential and largely achievable, particularly in larger facilities. However, real-time prescribing monitoring was deemed highly relevant but only moderately feasible due to system fragmentation and limited IT support. Pharmacy-led monitoring of Watch/Reserve antibiotics generated intense discussion around the practical 72 h restriction mechanism, with staff availability and digital tool gaps identified as main barriers. Alternative models such as trained link professionals and tele-AMS services were proposed for smaller facilities lacking dedicated personnel.

Community AMS was recognized as requiring adapted strategies distinct from hospital settings. Territorial multidisciplinary teams were strongly endorsed but required formal recognition and dedicated time allocation. Standardized clinical protocols for common infections were considered essential, with regional committees responsible for local guideline adaptation.

Education was identified as a transversal enabler cutting across all statements, with coordinated training initiatives and public awareness campaigns considered essential for sustainable implementation.

Detailed discussion points, perceived barriers, facilitating factors, and proposed reformulations for each statement are reported in Supplementary Table S1.

## 4. Discussion

Overall, the qualitative phase confirmed that governance integration, pharmacist leadership, data accessibility, and cross-regional learning are the key conditions participants identified for translating AMS strategies into sustainable practice. Participants reported a stronger sense of collective ownership and perceived feasibility after the structured, participatory workshop discussions, which the Steering Committee considered one of the most valuable aspects of the ARCO Step II methodology for turning theoretical recommendations into context-informed actions.

### 4.1. Interpretation of Findings

The ARCO Step II consensus identified a coherent set of priorities that together outline a multilevel governance model for sustainable AMS implementation. Fourteen high priority statements emerged across governance (1.1, 1.2, 1.3, 1.4, 1.6), hospital (2.1, 2.2, 2.5, 2.6), and community domains (3.1, 3.2, 3.4, 3.5, 3.6), representing the operational pillars of stewardship governance. These priorities align closely with the WHO Core Elements of Hospital AMS and with international frameworks emphasizing leadership commitment, multidisciplinary teams, tracking, training, standardizing and reporting as essential for stewardship effectiveness [2]. At the same time, the results highlight that the Italian context requires systemic embedding of AMS into healthcare governance rather than isolated programmatic initiatives.

The high consensus achieved around statements 1.1 and 1.2 confirms the perception that sustainability depends on linking stewardship outcomes to formal accountability mechanisms within regional and corporate performance systems: an approach consistent with recent European calls for governance-based stewardship [3,4]. Similarly, the strong endorsement of statements 2.5 and 2.6 demonstrates growing recognition of pharmacists as key operational leaders in AMS programs. These findings mirror evidence from the SAVE model and other European experiences where pharmacists’ involvement in monitoring restricted antibiotics, managing formularies, and providing feedback has proven to improve prescribing behavior and resource use [5,6].

The persistent challenges with digital infrastructure and data interoperability identified in our qualitative analysis reflect broader issues documented in Italian healthcare digitalization. In our study digitalization (statements 1.5, 2.3, and 3.3) received high relevance but lower feasibility scores; the discussions revealed a shared awareness that data integration and clinical informatics are indispensable for future progress. The emphasis placed by participants on data governance functions and standardized reporting formats mirrors recommendations from the European Joint Action on Antimicrobial Resistance and Healthcare-Associated Infections (EU-JAMRAI), which identified interoperable surveillance systems as essential infrastructure for effective AMS coordination [16].

The integration of quantitative and qualitative findings provided a comprehensive understanding of the consensus-building process, illustrating how multidisciplinary discussion and interregional exchange shaped the refinement and prioritization of AMS strategies across regional health systems.

The greater increase in feasibility scores (+0.78) compared to relevance (+0.44) following structured discussion suggests that perceived barriers to AMS implementation are often related to information gaps and lack of operational clarity, rather than fundamental disagreement on strategic priorities. This finding aligns with studies demonstrating that interactive deliberation and peer exchange can reduce implementation resistance by clarifying practical pathways and sharing contextual solutions [17,18]. The workshop format allowed participants to move from abstract policy recognition to concrete operational consensus, demonstrating the value of participatory approaches in stewardship governance.

### 4.2. Comparison with Existing Literature

The ARCO Step II results complement and extend the findings from ARCO Step I, which mapped the regional implementation of AMS and IPC programs in the Veneto Region [9]. While Step I demonstrated structural progress, it also revealed uneven operationalization and gaps in coordination between hospitals and community care. Step II builds on that evidence by testing a Delphi-based participatory model capable of translating strategic directions into shared, feasible actions. The approach resonates with the principles of the SPiNCAR model, which promotes standardized indicators for AMS governance evaluation [10], and with the GPPAS and MASPIC programs, which advocate for multidisciplinary stewardship interventions at the community level [15,19]. By combining the Delphi method with interregional participation, ARCO Step II provides a replicable framework for bridging institutional and operational dimensions of stewardship.

Our findings align with international Delphi studies on AMS implementation priorities. Mendelson et al. [20] identified leadership commitment and multidisciplinary team structures as fundamental enablers in low- and middle-income countries, while Stanić Benić et al. [21] emphasized the role of dedicated resources and clear accountability mechanisms in European settings. Similarly to our results, both studies found consensus more easily achieved on strategic goals than on operational feasibility, underscoring the persistent implementation gap documented across healthcare systems [22,23].

The high priority assigned to territorial multidisciplinary teams (Statement 3.1) and standardized community protocols (Statement 3.2) reflects growing recognition that AMR control requires coordinated action across the entire human-health care continuum. This hospital–community coordination is complementary to, but distinct from, the broader One Health perspective, which additionally encompasses animal and environmental dimensions of AMR not directly assessed in this study [24].

### 4.3. Methodological Contribution

Beyond the content of the consensus, the study contributes methodologically by demonstrating that structured, peer-to-peer deliberation across diverse organizational contexts can enhance the perceived feasibility of complex system-level strategies. The iterative combination of prework scoring, moderated round-table discussions, and final Delphi voting generated a measurable shift in feasibility and relevance perceptions, confirming the value of deliberative consensus as a tool for implementation planning.

This process also reinforced interprofessional collaboration by bringing together directors, pharmacists, infectious disease specialists and microbiologists—groups that often operate in parallel within the Italian health system. The ARCO Step II methodology thus functions not only as a research instrument but also as an implementation catalyst, fostering horizontal integration and cross-regional standardization of AMS approaches.

### 4.4. Implications for Policy and Practice

From a policy perspective, the findings suggest that embedding AMS governance into formal performance and accountability systems is essential for ensuring sustainability. Health authorities may consider incorporating core AMS indicators—such as team presence, formulary compliance, and antibiotic consumption metrics—into regional evaluation frameworks and funding mechanisms. At the operational level, hospitals and local health authorities should prioritize the formal establishment of AMS teams, ensure adequate pharmacist staffing, and invest in digital infrastructures enabling real-time monitoring and feedback loops. Furthermore, the consensus on territorial stewardship underscores the need to expand AMS beyond hospital boundaries. These actions collectively address the long-standing fragmentation of stewardship efforts in Italy and align with the objectives of the PNCAR 2022–2025, promoting an integrated hospital–community approach to antimicrobial governance [7].

### 4.5. Strengths and Limitations

A key strength of the ARCO Step II study lies in its interregional and multidisciplinary design, which brought together professionals from hospital and community settings across three distinct regional health systems. This composition allowed the consensus process to capture heterogeneous organizational perspectives while identifying shared, transferable priorities for AMS governance. The inclusion of clinicians, pharmacists, microbiologists, medical directors, and territorial professionals ensured that the proposed statements reflected both strategic and operational viewpoints, enhancing their real-world relevance.

Another major strength is the mixed-methods modified Delphi approach, which combined quantitative scoring with structured, face-to-face deliberation. The integration of an in-person workshop between Delphi rounds proved particularly valuable in reducing perceived feasibility barriers, as demonstrated by the greater increase in feasibility compared with relevance scores. This deliberative component goes beyond traditional survey-based Delphi designs, enabling contextual benchmarking, peer learning, and refinement of implementation pathways. As such, the ARCO Step II methodology functioned not only as a consensus-building tool but also as an implementation-oriented process.

Several limitations should be acknowledged. First, although the panel size (n = 33) is consistent with established Delphi methodology, the geographic focus on Northeastern Italy may limit direct generalizability to other Italian regions or international settings with different governance structures and resource availability. However, the transferability of the proposed framework lies in its focus on governance mechanisms and implementation principles rather than context-specific organizational models. Second, feasibility scores were collected immediately after the workshop, which may have introduced a degree of optimism or social desirability bias related to group interaction. While this effect cannot be excluded, it also reflects one of the study’s objectives: to explore whether structured multidisciplinary dialog can meaningfully reshape perceptions of feasibility. Future longitudinal evaluations are needed to assess whether these perceptions translate into sustained practice change.

Third, participants were selected through professional AMS and IPC networks on a voluntary basis; as response rates and reasons for non-participation were not tracked, the extent of self-selection cannot be quantified, but this recruitment approach likely introduced selection bias toward individuals already engaged and motivated in stewardship activities. As a result, the level of consensus observed may overestimate readiness compared with the broader healthcare workforce. Related methodological biases specific to the modified Delphi design should also be considered. The in-person round-table format, while valuable for contextualizing scores, may have made participants’ views more visible to peers than in a fully anonymous Delphi, creating potential for conformity bias (convergence toward the group’s apparent view) and for more vocal or senior participants to disproportionately influence the discussion despite the anonymity of the subsequent scoring; the consistent increase in feasibility scores after the workshop is compatible with genuine clarification of implementation pathways, but cannot fully exclude a degree of social desirability or conformity effect, and this should be considered when interpreting the magnitude of Round 2 changes. The panel composition is a further source of bias: community medicine was represented by a single general practitioner, with no pediatrician, long-term care clinician, or practicing community pharmacist, so the community-domain results may not fully capture the perspectives of primary-care and community pharmacy professionals. Finally, because the panel was drawn from three Northeastern Italian regions with a comparatively mature, Veneto-led AMS governance model, we expect the underlying governance principles identified here (leadership accountability, multidisciplinary teams, standardized indicators, hospital–community integration) to be broadly transferable across healthcare systems, since they reflect internationally recognized AMS core elements rather than context-specific arrangements. The specific operational thresholds (e.g., review cycles, restriction mechanisms), by contrast, were calibrated to the Northeastern Italian regulatory context and may need local adaptation before adoption elsewhere. In addition, the study assessed perceived relevance and feasibility rather than actual implementation or clinical outcomes; therefore, causal inferences regarding the impact of the proposed strategies on antibiotic use or resistance cannot be made at this stage.

### 4.6. Future Implications

Building on the ARCO Step II experience, future developments will focus on different strategic directions. First, we would like to expand the network to additional health authorities, enabling broader benchmarking across heterogeneous governance and organizational models and strengthening the national relevance of the consensus framework. Second, efforts will be directed toward the formal institutionalization of the network within regional and national AMR governance structures, ensuring continuity, accountability, and long-term sustainability beyond project-based initiatives. This could be ideally done through the establishment of a permanent, multidisciplinary think tank on antimicrobial stewardship, capable of integrating clinical, pharmaceutical, microbiological, managerial, and public health perspectives.

## 5. Conclusions

The ARCO Step II project shows that structured, interregional, and multidisciplinary consensus-building can generate a shared, practice-oriented set of recommendations linking antimicrobial stewardship policy and operational practice. However, as an expert-consensus exercise, any claim of a realized bridge between policy and practice awaits confirmation through implementation studies.

The findings underscore that the sustainability of AMS depends not solely on technical interventions, but also on their systematic integration into healthcare governance, accountability mechanisms, and organizational workflows. By explicitly linking leadership responsibility, multidisciplinary collaboration, data-driven monitoring, and education across the hospital–community continuum, ARCO Step II advances a coherent governance-oriented model of stewardship aligned with national and European AMR strategies.

The proposed statements are offered as recommendations, and the overall approach as a potentially transferable model, to support regional health authorities and healthcare organizations in planning coordinated, sustainable antimicrobial stewardship in decentralized health systems; their effectiveness in practice remains to be evaluated prospectively.

## Figures and Tables

**Table 1:** Descriptions of thematic domains and statements.

Short Statement Title	Full Statement
Governance and organization	
1.1—AMS objectives in CEOs’ performance mandates	Integrating Antimicrobial Stewardship (AMS) objectives into the performance mandates of Chief Executive Officers through binding regional targets represents a strategic lever for the effective and sustainable implementation of AMS programs at the organizational level (Bravo et al., 2022 [14]; Istituto Superiore Sanità, p. 140914 [7]; “Success of antimicrobial stewardship programs—it starts with leadership and accountability—Frances Garraghan, 2022 [15]” .
1.2—Core AMS indicators in evaluation systems	Defining a set of core AMS indicators to be integrated into performance evaluation and accreditation systems is a fundamental step to ensure accountability, sustainability, and a practical governance tool to support AMS actions.
1.3—Valorization of link professionals and AMS competencies	Promoting structured instruments such as mapping of clinical competencies, development of dedicated job descriptions, and implementation of targeted training programs is essential to identify, qualify, and strengthen the role of link professionals in AMS, particularly in settings with limited or no specialist resources.
1.4—Strengthening microbiological diagnostics	Strengthening microbiological diagnostics, including rapid and molecular methods, represents a priority investment and organizational goal to improve timeliness, accuracy, and appropriateness of therapeutic decisions.
1.5—Digital systems and data governance for AMS	Integrating digital tools and Clinical Decision Support Systems (CDSS) into corporate information systems, together with structured and interoperable data flows between hospital and community care, constitutes a prerequisite for proactive, appropriate, and traceable management of antibiotic therapy.
1.6—Integrated AMS–IPC governance model	Adopting a lean and integrated governance model that structurally connects AMS and IPC programs, hospital and community settings, and both public and accredited private sectors, can enhance coherence, efficiency, and continuity of stewardship actions. Medical and Health Directorates should play a coordinating role supporting multidisciplinary teams and operational levels.
Hospital AMS implementation	
2.1—Establishment of multidisciplinary AMS teams	Establishing a hospital-based multidisciplinary AMS team (including infectious disease specialists, microbiologists, pharmacists, and the medical directorate) with a formal operational mandate to perform audits, therapeutic reviews, consultative support, and educational activities is a key component of integrated antibiotic management at the ward level.
2.2—Updated and binding antibiotic formulary	Adopting a corporate antibiotic formulary, updated at least every six months, with binding prescribing guidance and specific references for empirical, targeted, and discharge therapy, can improve consistency and appropriateness in antibiotic prescribing.
2.3—Real-time monitoring of antibiotic prescribing	Implementing real-time systems for monitoring antibiotic prescribing appropriateness, integrated within institutional information systems, can facilitate timely and informed prescribing decisions through alerts, mandatory justification requests, and automated decision-support functions.
2.4—Retrospective review and feedback on prescriptions	Introducing structured and regular retrospective reviews of antibiotic prescriptions (e.g., periodic audits or weekly/monthly reviews), with systematic feedback to wards and traceable corrective actions, can promote continuous and demonstrable improvement of prescribing practices.
2.5—Active pharmacy monitoring of Watch/Reserve antibiotics	Active monitoring by hospital pharmacy services of antibiotics belonging to the WHO Watch and Reserve categories—combined with a restricted 72 h supply for empirical therapy—can prevent inappropriate continuation of initial treatments and reinforce AMS team engagement in early therapeutic reassessment.
2.6—Regular reporting of antibiotic use and resistance data	Ensuring the regular production and dissemination of quantitative and qualitative reports on antibiotic consumption (at least quarterly) and resistance profiles (at least biannually), including cumulative antibiograms by ward or facility, provides essential information to support stewardship and clinical decision-making.
Community and primary-care AMS implementation	
3.1—Multidisciplinary territorial AMS teams	Establishing a multidisciplinary territorial AMS team with coordination, operational support, and training functions can promote stewardship culture among primary and community healthcare professionals, including general practitioners, pediatricians, long-term care physicians, and community pharmacists.
3.2—Standardized procedures for common community infections	Developing and sharing standardized corporate procedures for the management of the most frequent community infections (e.g., respiratory, urinary, skin) can improve prescribing consistency and adherence to evidence-based guidelines.
3.3—Digital prescribing alerts for GPs and pediatricians	Integrating tools such as mandatory diagnostic coding and prescribing alerts based on the WHO Access, Watch, Reserve (AWaRe) antibiotic classification into general practitioners’ and pediatricians’ electronic prescribing systems can guide antibiotic selection and promote more appropriate prescribing behavior.
3.4—AMS indicators in contracts with primary-care physicians	Including specific indicators on antibiotic use appropriateness within corporate agreements and contracts with general practitioners and pediatricians can support monitoring and alignment of prescribing practices with stewardship principles.
3.5—Reporting and feedback on antibiotic use and resistance	Producing and sharing regular reports on antibiotic consumption (at least every four months) and local resistance data (at least annually), accompanied by structured feedback meetings with professionals, can enhance awareness and adherence to AMS strategies.
3.6—Training and public awareness campaigns	Implementing regular and structured educational initiatives and awareness campaigns for healthcare professionals (including community pharmacists and private physicians) and the general population is essential to strengthen the culture of prudent antibiotic use and AMR prevention.

**Table 2:** Results of Round 1 and Round 2 for all statements.

Statement	Median Relevance Round 1 (IQR)	Median Relevance Round 2 (IQR)	Δ Median Relevance Round 1–Round 2	Median Feasibility Round 1 (IQR)	Median Feasibility Round 2 (IQR)	Δ Median Feasibility Round 1–Round 2	Priority Level
1.1	9 (2)	10 (1)	1	8 (1)	9 (2)	1	High (9.5)
1.2	9 (2)	10 (1)	1	8 (1)	9 (1)	1	High (9.5)
1.3	9 (2)	9 (2)	0	7 (2)	8 (1)	1	High (8.5)
1.4	9 (2)	9 (2)	0	7 (2)	8 (1)	1	High (8.5)
1.5	9 (2)	9 (2)	0	6 (2)	6.5 (2)	0.5	Moderate (7.75)
1.6	9 (2)	10 (1)	1	6.5 (3)	7.5 (2)	1	High (8.75)
2.1	10 (1)	10 (0)	0	8 (1.5)	9 (1)	1	High (9.5)
2.2	9 (2)	9 (2)	0	8 (1.5)	9 (1)	1	High (9)
2.3	9 (1)	9 (1.5)	0	6 (3)	6.5 (2)	0.5	Moderate (7.75)
2.4	9 (2)	9 (1)	0	7 (2)	7.5 (2)	0.5	Moderate (8.25)
2.5	9 (2)	10 (1)	1	8 (2)	8.5 (2)	0.5	High (9.25)
2.6	9 (1)	10 (1)	1	8 (2)	9 (2)	1	High (9.5)
3.1	9 (2)	10 (1)	1	6 (3)	8 (3.5)	2	High (9)
3.2	9 (1)	9 (1)	0	8 (1.5)	9 (2.5)	1	High (9)
3.3	9 (2)	9 (3)	0	7 (2)	7 (1.5)	0	Moderate (8)
3.4	8.5 (1.5)	9 (2)	0.5	8 (1)	8 (2)	0	High (8.5)
3.5	9 (2)	10 (1)	1	8 (2.5)	9 (2)	1	High (9.5)
3.6	9 (2)	9.5 (1)	0.5	7.5 (1)	7.5 (1.5)	0	High (8.5)

Priority-level cells are shaded to aid interpretation: green indicates statements classified as high priority (combined score ≥ 8.5); yellow indicates moderate priority (combined score < 8.5). Note that statements 1.6, 3.4, and 3.6 reached the high-priority combined-score threshold despite a feasibility median below the 8.0 consensus cut-off (see Section 3.1 and Section 4.5 for discussion of this classification rule).

## Data Availability

The data presented in this study are available from the corresponding author upon reasonable request. Access to the individual expert responses is restricted because they may only be shared with the explicit consent of the respective participant.

## Supplementary Materials

The following supporting information can be downloaded at https://www.mdpi.com/article/10.3390/microorganisms14081811/s1, Table S1: Qualitative insights from the discussions for each statements discussed. Table S2: ACCORD (ACcurate COnsensus Reporting Document) reporting checklist for this study. For the ARCO II Working Group: Address Information of the ARCO II Working Group.

## Abbreviations

The following abbreviations are used in this manuscript:

AMS	Antimicrobial Stewardship
FVG	Friuli-Venezia Giulia
AMR	Antimicrobial resistance
PNCAR	National Action Plan on Antimicrobial Resistance
IPC	Infection Prevention and Control
ARCO	Approcci di Rete per il Contrasto all’Antimicrobico-Resistenza Ospedale–Territorio
