# Supplementary material for: Bridging the Gap Between Strategy and Implementation in Antimicrobial Stewardship: Insights from the ARCO Step II Interregional Delphi Consensus in Northeastern Italy
Source: Microorganisms. 2026 Aug 17;14(8):1811. doi: 10.3390/microorganisms14081811 (PMC13515528; doi:10.3390/microorganisms14081811)
Supplement: Supplementary file 1 [file microorganisms-14-01811-s001.zip › microorganisms-4475223-For the ARCO II Working Group.pdf]

For the ARCO II Working Group:
Luca Arnoldo, Department of Medicine, University of Udine, Udine, Italy; Accreditation, Quality, and Clinical Risk Unit, Friuli Centrale Healthcare University Trust, Udine, Italy
Richard Aschbacher Microbiology and Virology Unit, Azienda Sanitaria dell'Alto Adige, Bolzano, Italy.
Giancarlo Basaglia, Department of Microbiology, ASFO "Santa Maria degli Angeli" Hospital of Pordenone, Pordenone, Italy
Desirè Bastarolo, Hospital Pharmacy Unit, Cittadella and Camposampiero Hospitals, ULSS 6 Euganea Health Authority, Padova, Italy
Barbara Basso, Hospital Pharmacy, ASFO 'Santa Maria degli Angeli' Hospital of Pordenone, Pordenone, Italy
Elena Carrara, Division of Infectious Diseases, Department of Diagnostic and Public Health, University of Verona, Verona, Italy
Annamaria Cattelan, Infectious and Tropical Diseases Unit, Padua University Hospital, Padua, Italy
Francesca Ciraolo, Hospital Health Direction, ULSS 4 Veneto Orientale Health Authority, San Donà di Piave, Venice, Italy.
Silvia Cognolato, Department of Pharmacy, Veneto Institute of Oncology IOV IRCCS, Padua, Italy
Cristina Contessa, Hospital Health Direction, ULSS 5 Polesana, Rovigo, Italy.
Mariateresa Gallea, General Practitioner, ULSS 6 Euganea Health Authority, Padova, Italy
Luca Martini, Infectious Diseases Division, Department of Medicine (DMED), University of Udine and Azienda Sanitaria Universitaria Friuli Centrale (ASUFC), Udine, Italy
Marta Mascarello, Infectious Diseases Unit, S. Bortolo Hospital, Vicenza, Italy
Annarita Mazzariol, Unit of Microbiology, University of Verona Hospital Trust, Verona, Italy
Andrea Misin, Infectious Disease Unit, Trieste University Hospital, Trieste, Italy
Leonardo Pagani, Infectious Diseases Unit, Central Hospital of Bolzano-Bozen, Bolzano, Italy
Roberta Rampazzo, Hospital Pharmacy, ULSS 5 Polesana Health Authority, St. Maria della Misericordia Hospital, Rovigo, Italy
Riccardo Roni, Primary Care Pharmaceutical Department, Azienda sanitaria universitaria integrata del Trentino, Trento, Italy
Alessia Salvador, Hospital Pharmacy Unit, All'Angelo Hospital, Mestre, Venice, Italy.
Claudio Scarparo, Unit of Microbiology, Azienda sanitaria universitaria integrata del Trentino, Trento, Italy
Federica Schievenin, Primary Care Pharmaceutical Department, ULSS 1 Dolomiti Health Authority, Belluno, Italy
Susanna Zardo, Healthcare District Nr.2, ULSS 3 Serenissima Health Authority, Venice, Italy
Gianluca Zuglian, Infectious Disease Unit, All'Angelo Hospital, Mestre, Venice, Italy
