# Supplementary material for: Bridging the Gap Between Strategy and Implementation in Antimicrobial Stewardship: Insights from the ARCO Step II Interregional Delphi Consensus in Northeastern Italy
Source: Microorganisms. 2026 Aug 17;14(8):1811. doi: 10.3390/microorganisms14081811 (PMC13515528; doi:10.3390/microorganisms14081811)
Supplement: Supplementary file 1 [file microorganisms-14-01811-s001.zip › microorganisms-4475223-supplementary.pdf]

SUPPLEMENTARY MATERIAL

Supplementary Table S1. Qualitative insights from the discussions for each statements discussed.

Statements	Qualitative insights from the discussions
1. Governance and organization	
1.1 – AMS objectives in CEOs' performance mandates	Participants unanimously recognized the inclusion of AMS objectives in the performance mandates of Chief Executive Officers as a structural driver for stewardship sustainability. This linkage was seen as a necessary precondition for institutional accountability and for ensuring managerial engagement beyond clinical departments. Regional representatives highlighted that existing systems (e.g., Nuovo Sistema di Garanzia) could already host AMS indicators, provided that they are clearly defined and measurable.
1.2 – Core AMS indicators in evaluation systems	The discussion focused on defining a minimal, standardized set of AMS indicators suitable for integration into performance and accreditation systems. Participants emphasized the need for uniformity across regions to enable benchmarking and transparency. It was suggested that indicators should combine process (e.g., presence of AMS teams, reporting frequency) and outcome measures (e.g., antibiotic consumption, resistance trends).
1.3 – Valorization of link professionals and AMS competencies	This statement was widely endorsed. Participants discussed the diversity of professional figures who can act as link professionals (nurses, pharmacists, physicians) and the importance of structured role definition, recognition, and dedicated training. Some regions already adopted local mapping tools for AMS competencies; others identified this as an urgent need, particularly in smaller hospitals and community facilities.
1.4 – Strengthening microbiological diagnostics	All groups confirmed the central role of timely microbiological data for stewardship effectiveness. The main barriers identified were the shortage of laboratory staff, delayed reporting, and uneven access to rapid or molecular diagnostics. Participants proposed prioritizing investments in diagnostic platforms and strengthening communication channels between microbiology services and AMS teams.
1.5 – Digital systems and data governance for AMS	The statement was considered strategically relevant but challenging to implement. Participants reported wide variability in digital maturity across regions and facilities. The lack of interoperability between hospital and territorial databases was a recurrent issue. Proposals included the creation of data stewardship functions to ensure data standardization and the progressive integration of prescribing and microbiological information systems.
1.6 – Integrated AMS–IPC governance model	This was one of the most debated statements. Participants strongly supported the need for integrated governance models linking AMS and IPC at both hospital and territorial levels. Examples from Veneto and Friuli Venezia Giulia—where AMS and IPC activities are

	coordinated by the Medical Directorate—were cited as effective models. It was suggested that joint reporting and shared operational plans could enhance efficiency and consistency.
2. Hospital AMS implementation	
2.1 – Establishment of multidisciplinary AMS teams	There was full consensus on the importance of establishing formally recognized multidisciplinary AMS teams including infectious disease specialists, pharmacists, microbiologists, and medical directors. In large hospitals, such teams were already active; in smaller facilities, the lack of dedicated personnel was reported as a key limitation. Participants discussed alternative models based on trained link professionals or remote support through tele-AMS services.
2.2 – Updated and binding antibiotic formulary	Participants agreed that a corporate antibiotic formulary is essential to harmonize prescribing practices and reduce variability across departments. Several hospitals reported positive experiences with electronic formularies linked to prescribing systems. The group proposed that formularies be updated at least every six months and validated by both AMS teams and clinical departments.
2.3 – Real-time monitoring of antibiotic prescribing	The statement was perceived as highly relevant but only moderately feasible. Participants noted that while the technical capability exists in some hospitals, system fragmentation and limited IT support hinder implementation. Pilot projects using dashboards and real-time alerts were presented as promising examples, but scalability remains constrained.
2.4 – Retrospective review and feedback on prescriptions	This statement was considered feasible and already applied in various forms. Participants described regular audits and case reviews as valuable for continuous improvement and professional engagement. The need for structured documentation of feedback and follow-up actions was highlighted to ensure traceability.
2.5 – Active pharmacy monitoring of Watch/Reserve antibiotics	This topic generated intense discussion. All participants recognized the value of pharmacy-led monitoring to improve antibiotic appropriateness. The 72-hour restriction for empirical therapy was identified as a practical mechanism to trigger AMS review. Barriers included staff availability and the absence of digital prescription management tools in some facilities. The statement was ultimately considered realistic if implemented within the framework of multidisciplinary AMS teams.
2.6 – Regular reporting of antibiotic use and resistance data	There was unanimous agreement on the importance of structured reporting. Participants emphasized the need to standardize the frequency (quarterly for consumption, biannual for resistance) and format of reports, and to ensure feedback reaches all relevant professionals. Some regions already produce integrated corporate reports combining hospital and community data.
3. Community and primary-care AMS	

3.1 – Multidisciplinary territorial AMS teams	The statement received strong consensus. Participants described ongoing experiences where district-level teams include pharmacists, infectious disease specialists, and general practitioners. Such teams were viewed as key to coordinating training, data analysis, and communication between hospitals and community care. The main limitation identified was the lack of formal recognition and dedicated time.
3.2 – Standardized procedures for common community infections	Participants agreed on the need for standardized clinical protocols for the most frequent infections managed in primary care. These tools should be concise, evidence-based, and shared across regions to ensure consistent prescribing behavior. The discussion highlighted the role of regional scientific committees in adapting national guidelines to local contexts.
3.3 – Digital prescribing alerts for GPs and pediatricians	This statement generated mixed views. While participants agreed on its potential to improve appropriateness, concerns were raised about the heterogeneity of prescribing software and the risk of alert fatigue. Some proposed introducing diagnostic justification prompts as a first step before full alert systems.
3.4 – AMS indicators in primary-care contracts	The inclusion of AMS indicators in agreements with general practitioners and pediatricians was considered an effective but complex mechanism to promote accountability. Participants suggested starting with a limited set of indicators (e.g., antibiotic consumption per 1000 patients, use of Watch antibiotics) and integrating them into existing monitoring systems
3.5 – Reporting and feedback on antibiotic use and resistance	This statement was supported unanimously. Participants from Friuli Venezia Giulia described their experience with periodic feedback reports to general practitioners as a replicable model. Regular reporting was viewed as an accessible and effective method to engage prescribers and monitor trends over time.
3.6 – Training and public awareness campaigns	Education was identified as a transversal enabler for all other statements. Participants proposed coordinated training initiatives targeting general practitioners, pharmacists, and nursing home staff, combined with public awareness campaigns. Several regions already conduct joint sessions or local campaigns promoting rational antibiotic use and AMR prevention.

Supplementary Table S2. ACCORD reporting checklist for the ARCO Step II consensus study

ACCORD (ACcurate COnsensus Reporting Document): Gattrell WT, Logullo P, van Zuuren EJ, et al. PLOS Medicine 2024;21(1):e1004326 (doi:10.1371/journal.pmed.1004326). Explanation and elaboration: Logullo P, van Zuuren EJ, Winchester CC, et al. PLOS Medicine 2024;21(5):e1004390 (doi:10.1371/journal.pmed.1004390).

IMPORTANT – read before use: rows shaded green reproduce the official ACCORD item wording, verified directly against the ACCORD Explanation & Elaboration document (items T1, I1–I3, M1–M15; 16 of 35 items). Rows shaded yellow could not be verified against the official checklist text in this session (the source was only available as a non-extractable image/behind a rate limit) and instead describe the general topic area covered by that section, inferred from the published item counts (Methods n=21, Results n=5, Discussion n=2, Other n=3) and partial confirmation in the guideline's own Results section. Before submission, the corresponding author should download the official ACCORD checklist (Word/PDF) from the EQUATOR Network (https://www.equator-network.org/reporting-guidelines/accord/) or the PLOS Medicine supporting information (S9 Text of doi:10.1371/journal.pmed.1004326) and replace the yellow rows with the verbatim item wording before this table is submitted as Supplementary Table S2.

Item	ACCORD checklist item	Status	Reported in manuscript
**T1**	Identify the article as reporting a consensus exercise and state the consensus methods used in the title.	Confirmed wording	Title
**I1**	Explain why a consensus exercise was chosen over other approaches.	Confirmed wording	Introduction, para. 1–2
**I2**	State the aim of the consensus exercise, including its intended audience and geographical scope (national, regional, global).	Confirmed wording	Introduction, final paragraph (objectives)
**I3**	If the consensus exercise is an update of an existing document, state why an update is needed, and provide the citation for the original document.	Confirmed wording	Not applicable – ARCO Step II is not an update of a prior document (it builds on, but does not replace, ARCO Step I)
**M1**	If the study or study protocol was prospectively registered, state the registration platform and provide a link. If not registered, state this.	Confirmed wording	Not prospectively registered – to be stated explicitly in Methods
**M2**	Describe the role(s) and areas of expertise or experience of those directing the consensus study.	Confirmed wording	Methods, Steering Committee paragraph
**M3**	Explain the criteria for panellist inclusion and the rationale for panellist numbers. State who was responsible for panellist selection.	Confirmed wording	Methods, participant-selection paragraph
**M4**	Describe the recruitment process (how panellists were invited to participate).	Confirmed wording	Methods, participant-selection paragraph
**M5**	Describe the role of any members of the public, patients, or carers in the different steps of the study.	Confirmed wording	Not applicable – no patient/public panellists; to be stated explicitly
**M6**	Describe how information was obtained prior to generating items or other materials used during the consensus exercise.	Confirmed wording	Methods, statement-development paragraph (literature, policy frameworks, ARCO Step I)
**M7**	Describe any systematic literature search in detail (search strategy, dates) or cite if published.	Confirmed wording	Not a systematic review – narrative literature/policy review only; to be stated explicitly

Item	ACCORD checklist item	Status	Reported in manuscript
**M8**	Describe how any existing scientific evidence was summarised and if this evidence was provided to the panellists.	Confirmed wording	Methods, pre-work phase (18 draft statements sent to panellists)
**M9**	Describe the methods and steps used to gather panellist input and reach consensus (e.g., Delphi, RAND-UCLA, NGT). Report modifications.	Confirmed wording	Methods, consensus-methodology paragraph (modified Delphi)
**M10**	Describe how each question/statement was presented and the response options; whether panellists could explain responses or propose new items.	Confirmed wording	Methods, statement-presentation paragraph; Supplementary File (statement list)
**M11**	State the objective of each consensus step (meeting, discussion/interview session, Delphi round).	Confirmed wording	Methods; Figure 1 (process flowchart)
**M12**	State the definition of consensus (number, percentage, or categorical rating) and the rationale for that definition.	Confirmed wording	Methods, consensus-threshold paragraph (expanded with literature justification)
**M13**	State whether items that met the pre-specified definition of consensus were included in subsequent voting rounds.	Confirmed wording	Methods – all 18 statements were re-scored in Round 2 regardless of Round 1 consensus; to be stated explicitly
**M14**	For each step, describe how responses were collected, and whether individually or in a group setting.	Confirmed wording	Methods (individual scoring pre/post workshop) and Results (round-table discussion format)
**M15**	Describe how responses were processed and/or synthesised (quantitative and/or qualitative methods).	Confirmed wording	Methods, statistical-analysis and qualitative-analysis paragraphs
**M16–M21**	Remaining Methods items (6 items) – ACCORD Methods section covers 21 items in total; items M16–M21 typically address topics such as anonymity/blinding of responses, handling of missing data or panellist attrition, ethics approval, and statistical methods for synthesising quantitative results.	Topic area – not verified	Partially addressed in Methods (attrition not applicable – same 33 panellists scored both rounds; ethics: no ethics committee review was sought, as is common for non-patient professional consensus panels – to be stated explicitly)
**R1–R2**	Results items (2 of 5) – typically cover panellist participation/response rate per round and panellist characteristics.	Topic area – not verified	Reported in Methods/Results (33/33 panellists, no dropout between rounds; panel composition in Methods)
**R3**	Report the quantitative results of the consensus process (confirmed topic: quantitative data from voting rounds).	Topic area – not verified	Results, Section 3.1; Table 2
**R4**	Report the qualitative results of the consensus process (confirmed topic: qualitative data from voting rounds).	Topic area – not verified	Results, Section 3.2; Supplementary Table S1
**R5**	Report any modifications made to items/topics during the consensus process (confirmed topic, combines original items on mid-process changes).	Topic area – not verified	Methods/Results – statements were reformulated during the workshop; to be cross-referenced explicitly
**D1–D2**	Discussion items (2 items) – ACCORD Discussion section typically requires interpretation of findings in context and an explicit discussion of strengths and limitations, including potential biases.	Topic area – not verified	Discussion, Section 4 (interpretation) and Section 4.5 (expanded strengths/limitations and bias discussion)

Item	ACCORD checklist item	Status	Reported in manuscript
**O1–O3**	Other information items (3 items) – typically cover funding/role of funders, conflicts of interest, and data/protocol availability.	Topic area – not verified	Manuscript back matter (Funding, Conflicts of Interest, Data Availability Statement already present)
